# T cell exhaustion: a two-sided blade in systemic lupus erythematosus, from molecular mechanisms to clinical translation

**DOI:** 10.3389/fimmu.2026.1871551

**Published:** 2026-07-10

**Authors:** Yanwei Bi, Feifeng Wu, Jueyi Mao, Tasnim Azad, Pengcheng Liu, Xin Zhou, Haotian Xie, Kimsor Hong, Xinying Qiu, Binbin Li, Miaomiao Wu, Liang Zhang, Jidong Tian, Chuan Wen

**Affiliations:** 1 Department of Pediatrics, The Second Xiangya Hospital of Central South University, Changsha, China; 2 The Affiliated Children’s Hospital of Xiangya School of Medicine, Central South University, Changsha, China; 3 Department of Nephrology, Rheumatology and Immunology, Hunan Children’s Hospital, The Paediatric Academy of University of South China, Changsha, China; 4 Department of Gastroenterology, The Second Xiangya Hospital of Central South University, Changsha, China

**Keywords:** autoimmune diseases, bidirectional regulation, systemic lupus erythematosus, T cell exhaustion, targeted therapy

## Abstract

Dysregulated immune responses and extensive inflammatory damage to several organs are hallmarks of systemic lupus erythematosus (SLE), a highly heterogeneous systemic autoimmune disease that significantly impairs patients’ quality of life and prognosis. Persistent antigenic stimulation causes T cell exhaustion (Tex), a unique functional state that is carefully controlled by exogenous, temporal, and spatial factors. Tex plays a special bidirectional regulatory role in SLE: on the one hand, it exerts a protective effect by suppressing the excessive activation of autoreactive T cells, thereby mitigating immune-mediated damage to target organs; on the other hand, abnormal exhaustion of specific T cell subsets leads to a loss of immunoregulatory function and may even promote the secretion of pro-inflammatory cytokines, thereby exacerbating pathological damage. Therapeutic strategies based on this dual mechanism focus on restoring immune homeostasis by enhancing protective exhaustion and inhibiting pathogenic exhaustion processes; this can be achieved through precise regulation of exhausted T cell subsets, improvement of the immune microenvironment, and interventions targeting key signalling pathways. This article provides a systematic review of the core molecular mechanisms and phenotypic characteristics of Tex, highlighting the latest research advances regarding its bidirectional regulatory role in SLE, and discusses in detail relevant diagnostic biomarkers and potential targeted therapeutic strategies. It aims to provide new insights and a theoretical basis for the clinical assessment, personalised treatment, and targeted drug development of SLE.

## 1 Background

**Figure 1:**
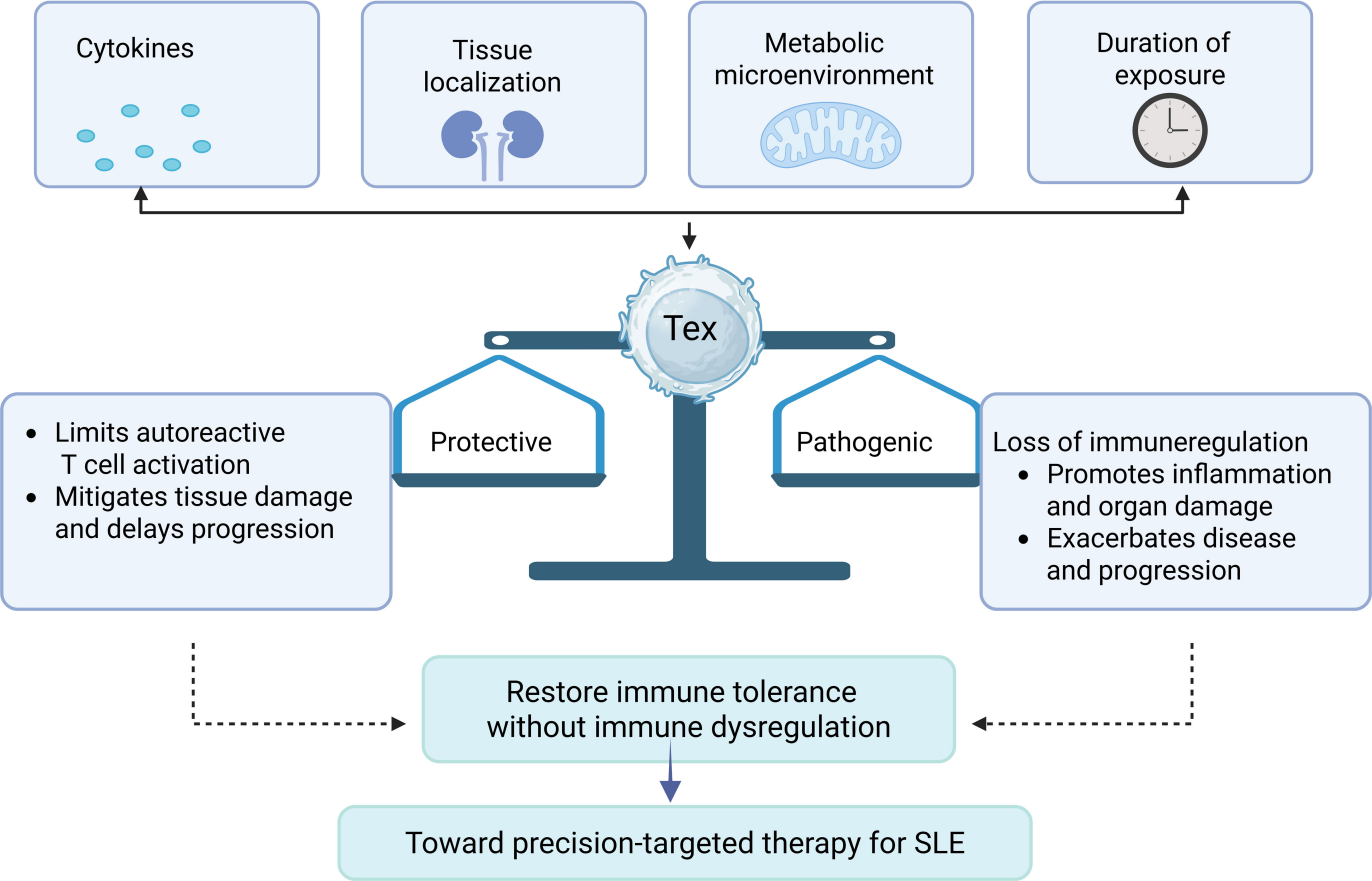
The Balance of Protection and Pathogenicity: Disruption and Reestablishment of Immune Tolerance in SLE.

Systemic lupus erythematosus (SLE) is a highly heterogeneous autoimmune disorder characterised by multi-organ damage resulting from the aberrant activation of autoreactive lymphocytes following the breakdown of immune tolerance (1). Traditional immunosuppressants and biologics frequently encounter challenges such as inadequate efficacy, high recurrence rates and significant adverse effects (2, 3). T cells, as the principal constituents of the adaptive immune system, directly regulate the magnitude and specificity of immune responses via their functional status. T cell exhaustion (Tex) was initially identified in chronic infections and tumour microenvironments, marked by reduced effector function and elevated expression of immune checkpoint molecules (4). In SLE, continuous self-antigen stimulation progressively induces Tex, regulated by the cumulative influence of external factors such as the duration of antigen exposure, tissue localisation, cytokines, and the metabolic microenvironment (5). Moderate Tex may delay disease progression by suppressing excessive immune responses, whereas abnormal depletion of specific subsets disrupts immune homeostasis and exacerbates pathological damage. This duality makes it a key starting point for understanding how SLE develops (6–8). With the development of metabolomics, epigenetics and single cell sequencing technology, new mechanisms of exhaustion regulation such as NFAT1 activation mediated by methionine metabolism (9, 10) and chromatin loop remodelling regulated by Interferon Regulatory Factor 8(IRF8)have been gradually revealed (10). At the same time, finding biomarkers like MX1 and terminal uridyltransferase (TUTase) has given us objective tools for figuring out how severe a disease is and how well treatments are working (11, 12). Given the dual-action nature of Tex, targeting strategies have shifted from conventional non-specific immunosuppression towards precise regulation. This approach enhances protective exhaustion whilst suppressing pathogenic exhaustion, thereby restoring immune tolerance whilst avoiding immune dysregulation caused by excessive intervention. This paper systematically clarifies the fundamental regulatory features of Tex, emphasising its dual mechanisms in SLE, related biomarkers, and targeted therapeutic strategies. It seeks to offer innovative insights into the elucidation of SLE pathogenesis and establish a foundation for the development of highly effective, precision-targeted therapeutic strategies. We’ve summarised the above in **Figure 1**.

## 2 Mechanistic induction of Tex and their association with SLE

### 2.1 Three key characteristics of Tex

Tex is a distinct state of T cell dysfunction induced by persistent antigen stimulation and is commonly observed in chronic infections, cancer, and autoimmune diseases. Recent studies suggest that T cell subsets resembling exhausted T cells are also present in SLE, where they may contribute to immune regulation and disease heterogeneity. The development of Tex is shaped by temporal, spatial, and environmental factors that collectively influence T ell fate decisions.

#### 2.1.1 Temporal aspects

Persistent antigen exposure is the central driver of Tex. Continuous T cell receptor (TCR) signalling induces progressive loss of effector function, sustained expression of inhibitory receptors, and the establishment of exhaustion-associated transcriptional programmes (13, 14).

In SLE, chronic exposure to self-antigens, including nuclear antigens and immune complexes, provides a persistent stimulatory environment that may promote the development of a T cell state resembling exhaustion. This process is thought to represent an adaptive mechanism limiting excessive immune activation, although it may also impair protective immune responses. It is worth noting that exhaustion progresses from stem-like progenitor cells to terminally exhausted cells through a hierarchical differentiation process (5, 15).

#### 2.1.2 Spatiality

Lymphoid tissues provide a supportive microenvironment for the maintenance of exhausted T cell progenitor cells. These cells retain the potential for self-renewal and serve as a reservoir for subsequent differentiation (16). Following migration into peripheral tissues, exhausted T cells are exposed to sustained antigenic stimulation and inflammatory signals that promote terminal differentiation (17). In autoimmune diseases such as systemic lupus erythematosus, secondary lymphoid organs may similarly support the persistence of chronically stimulated autoreactive T cells, although the exact anatomical distribution of Tex cell populations remains unclear.

#### 2.1.3 Exogenous

Cytokines play a critical role in regulating the differentiation and maintenance of exhausted T cells. Type I interferons, which are central mediators of SLE pathogenesis, have been shown to reduce T cell stemness and promote exhaustion-associated transcriptional programmes. Conversely, TGF-β may preserve progenitor exhausted T-cell populations through regulation of the BCL6 and mTOR pathways whilst simultaneously promoting inhibitory receptor expression (18–20). Given the prominent interferon signature observed in SLE, cytokine-driven regulation of Tex may represent an important mechanism linking chronic inflammation to T cell dysfunction (21).

### 2.2 Emerging regulatory mechanisms of Tex and their potential relevance to SLE

Recent studies in chronic infection and cancer have revealed that Tex is regulated by multiple layers of control extending beyond persistent antigen stimulation. These include metabolic reprogramming, epigenetic remodelling, microbiota-derived metabolites and protein homeostasis pathways. Although most evidence originates from tumour immunology, these mechanisms may provide valuable insights into the development and maintenance of T cell dysfunction in SLE, where chronic autoantigen exposure and sustained immune activation are also prominent features.

#### 2.2.1 Metabolic regulation of Tex

Cell metabolism has been shown to be a key determinant of T cell fate. Methionine metabolism regulates methylation-dependent transcriptional programmes by modulating the intracellular availability of S-adenosylmethionine. In tumour models, methionine deficiency promotes the expression of exhaustion-associated molecules such as PD-1 and TIM-3 by altering calcium signalling and epigenetic regulation (9). Although direct evidence in SLE remains limited, metabolic abnormalities involving mTOR activation, amino acid utilisation and mitochondrial dysfunction have been identified as hallmarks of lupus T cells. Given the central role of methionine metabolism in regulating T cell activation and differentiation, future research should investigate whether similar pathways are involved in the formation of exhausted T cell subsets in SLE (22).

Recent studies have also shown that different pathways for the utilisation of acetyl-CoA influence the balance between progenitor cells and terminally exhausted CD8^+^T cells via histone acetylation (23). It remains to be determined whether a similar metabolic-epigenetic coupling is involved in the formation of the heterogeneous exhausted T cell populations observed in SLE.

#### 2.2.2 Epigenetic regulation of Tex

A growing body of evidence suggests that Tex is accompanied by stable epigenetic reprogramming. Transcription factors such as IRF8 and SATB1 act in concert with chromatin modification complexes to establish a transcriptional landscape specific to exhaustion. These mechanisms regulate the accessibility of genes involved in the expression of inhibitory receptors, cytokine production and cell differentiation (10, 24). Given that SLE is characterised by widespread epigenetic dysregulation, including low DNA methylation and altered histone modifications in T cells, these findings suggest that epigenetic regulatory networks associated with exhaustion may also be involved in the pathogenesis of lupus (25).

In summary, these emerging mechanisms suggest that Tex is regulated by an integrated network involving metabolic, epigenetic and proteostatic signalling. Understanding how these pathways function in SLE may help identify novel biomarkers and therapeutic targets aimed at restoring immune balance whilst avoiding excessive immune activation.

### 2.3 Unique characteristics of Tex in autoimmune disease settings

In the context of autoimmune diseases, the dual effects of this state are especially evident. It can either limit tissue damage caused by self-reactive T cells and slow the progression of disease by suppressing overactive immune responses, or it can cause damage by causing functional problems in certain exhausted T cell subsets, which makes it a key target for studying the causes of autoimmune disease and how to treat it. Continuous self-antigen stimulation can lead CD4^+^and CD8^+^T cells into states of progressive exhaustion. These exhausted T cells exhibit core characteristics akin to those of exhausted T cells in tumours or chronic infections, including elevated expression of inhibitory receptors such as PD-1, LAG-3 and TIM-3. In contrast, CD4^+^ and CD8^+^Tex display a shared co-inhibitory gene expression pattern, whilst also presenting distinct molecular signatures associated with autoimmune diseases. For example, most CD4^+^ effector T cells that infiltrate islets in type 1 diabetes models keep TCF1 expression, which keeps their ability to grow and secrete IFNγ. This is different from CD4^+^ T cells in chronic infections, which quickly become terminally exhausted. This phenotype stays the same as the disease gets worse (26). It is important to note that CD4^+^ Tex usually keeps some immunoregulatory or helper functions, but these functions are usually weaker or not working properly (27, 28).

From the standpoint of pathological mechanisms, the bidirectional regulatory effects of Tex on autoimmune diseases are evident in conditions like type 1 diabetes and neuromyelitis optica spectrum disorders, where the phenotypic characteristics of Tex cells are intricately associated with disease progression. Moderate depletion of these cells provides a protective mechanism by curbing excessive immune responses (26, 29); analogous to findings in cancer research, the TCF1^+^CD8^+^ T cell precursor population primarily localises in tumour-draining lymph nodes, demonstrating significant stemness, plasticity, and self-renewal potential (30). When this group is brought into the tumour microenvironment, it slowly moves towards an exhausted state (15). This mechanism offers essential insights into the differentiation patterns of Tex cells in autoimmune diseases.

The control of Tex in autoimmune diseases operates through a synergistic interplay between the “external microenvironment and intrinsic molecular network.” At the external regulatory level, chronic self-antigen stimulation, a dysregulated cytokine milieu including pleiotropic cytokines such as IL-2 and IL-10, sustained activation of co-inhibitory receptors such as PD-1 and CTLA-4 and metabolic microenvironment abnormalities, including lactic acid accumulation and glucose deprivation, collectively facilitate the exhaustion process (5). At the intrinsic level, dysregulation of the TCF1-BCL6-BLIMP regulatory circuit constitutes the fundamental intrinsic mechanism, directing CD8^+^ T cells towards either progenitor or terminally differentiated states (18, 31); Abnormal activation of transcription factors such as TOX and NR4A directly induces Tex by entrapping cells in a dysfunctional state via the upregulation of inhibitory receptors and the suppression of effector genes (5, 18, 31). These transcription factors create a synergistic exhaustion network by working together to promote and stabilise the exhausted phenotype.

## 3 The core relationship between T cell exhaustion and systemic lupus erythematosus: a double-edged sword of bidirectional regulation

Tex functions as a double-edged sword in SLE, demonstrating bidirectional regulatory effects that are simultaneously protective and pathogenic. Its functional orientation is not static; it is collaboratively influenced by the attributes of Tex subsets (e.g., CD8^+^ T cell subsets, regulatory T cell subsets), the local tissue microenvironment (e.g., inflammatory cytokine concentrations, metabolic levels, antigen exposure intensity), and molecular regulatory networks (e.g., PD-1/PD-L1 signalling, and the TCF1-BCL6 pathway). This makes it a key place to start figuring out how autoimmune diseases start and where to look for ways to treat them (31). Moderate Tex can protect target organs like the central nervous system from immune damage by stopping overactive autoreactive T cells from working. For example, tired CD8^+^T cells in the choroid plexus of mice with neuropsychiatric lupus stop cytotoxic function to protect the CNS from damage (8). In terms of pathogenic effects, functional abnormalities in certain exhausted subpopulations disturb immune homeostasis. For example, CCR7^low^CD74^hi^ regulatory T cells in patients with SLE display an exhausted phenotype, resulting in the loss of their inhibitory function and the promotion of autoimmunity (6). This duality establishes Tex as a fundamental aspect necessitating meticulous regulation in autoimmune disease research.

### 3.1 Protective role—suppressing excessive immune responses and delaying disease progression

A recent clinical trial revealed that CD4^+^EOMES^+^cells are also elevated in long-term remission SLE patients compared to those with active disease. In remission patients, CD4^+^EOMES^+^T cells exert a protective effect by suppressing excessive immune responses to maintain disease stability. Compared to healthy subjects, SLE patients exhibit increased expression of PD-1, CD57 and EOMES on both exhausted and activated T cells. These markers may thus serve as potential indicators of disease remission. This suggests that the accumulation of these exhaustion-associated phenotypes may represent a crucial mechanism for establishing immune tolerance and maintaining disease remission. Depleting T cells in specific populations could be a potential therapeutic tool to help achieve sustained remission in these patients (7).

In line with this discovery, specific CD8^+^ regulatory T cell subsets provide protective benefits in lymph nodes by diminishing autoreactive B cells and enhancing immunoregulation, thereby increasing the overall complexity of the scenario (32, 33). Moreover, LAG-3 expressing regulatory T cells inhibit B cell responses through TGF-β3 secretion in a PD-1 dependent manner; however, the prevalence of these protective Tregs is diminished in SLE patients (34).

Notably, a recent study focusing on the choroid plexus (ChP)—a core component of the blood-cerebrospinal fluid barrier (BCSFB)—characterised T cell subsets infiltrating the ChP in neuropsychiatric lupus (NPSLE) mice. Depletion-associated genes were primarily enriched in CD8^+^T cells. In aged (late-stage disease) MRL/lpr mice, ChP-infiltrating T cells demonstrated significantly elevated expression of exhaustion-associated genes relative to young (early-stage disease) mice, accompanied by markedly diminished TCR clonal diversity and pronounced local proliferative characteristics. However, compared to young LPR mice, aged LPR mice showed significantly decreased expression of cytotoxic-related genes in choroid plexus T cells. This phenomenon suggests that as disease progresses, T cell exhaustion may represent an adaptive response to chronic antigenic stimulation, such as the CNS-specific antigen MBP. By reducing T cell hyperactivation and suppressing cytotoxic function, this mechanism prevents sustained CNS tissue attack, thereby partially limiting progression to more severe neurological damage (8).

The study interestingly compared ChP-infiltrating T cells to those infiltrating the kidney and spleen, showing that ChP T cells were more exhausted and had much higher levels of hypoxia-related genes (8). In sharp contrast to renal infiltrating T cells, peripheral blood from LN patients exhibited significantly elevated exhausted PD-1^+^CD4^+^ and CD8^+^ T cell subsets. Nonetheless, PD-1^+^CD8^+^T cells infiltrating the kidney were not functionally exhausted; rather, they represented a highly activated, clonally expanded, and cytotoxic population, with their enrichment correlating with more severe renal pathology. The difference in T cell states between circulation and tissues suggests that the renal microenvironment provides continuous antigenic stimulation that keeps T cells active instead of making them tired. Activating the PD-1/PD-L1 pathway, like with PD-L1 Fc therapy, can modulate the functional state of PD-1^+^CD8^+^T cells through the Stat1-T-bet-IFN-γ axis. This can make LN disease activity better (35, 36). In contrast, T cells infiltrating the choroid plexus exhibit a strongly exhausted, low-toxicity phenotype. This discrepancy suggests that at the choroid plexus—a central nervous system barrier—T cell exhaustion may serve a protective function by limiting immune damage, rather than the complex coexistence of exhaustion and injury observed in renal tissue. This indirectly supports its potential to delay neurodegenerative disease progression (8).

### 3.2 Reverse pathogenic effects—promoting autoimmunity and driving pathological damage

Regulatory CD4^+^T cells are a recognised subset of CD4^+^T cells that play a vital role in sustaining immune tolerance, essential for immune system homeostasis. It is established that CD4^+^T cells facilitate the progression of SLE (37). A recent human study utilising high-throughput sequencing (HTS) and single-cell RNA sequencing (scRNA-seq) to examine peripheral blood CD4^+^T cells from SLE patients and healthy controls further elucidated transcriptional dysfunction in regulatory T cells. It identified two Treg subsets, with CCR7^low^CD74^hi^Treg cells in SLE patients demonstrating type I IFN-associated functional exhaustion and enduring dysregulation patterns in transcriptomes and chromatin accessibility. This indicates that they not only forfeit their suppressive function but also develop an exhaustion-like phenotype that may, paradoxically, facilitate autoimmunity. Interestingly, very similar Tregs were found in CD4^+^T cell datasets from patients with ulcerative colitis and multiple sclerosis. However, differences in antigens may explain the different levels of exhaustion, which need to be confirmed by antigen-specific T cell sorting (6).

In SLE mouse models, double-negative T cells (DNT, CD4^−^CD8^−^) were more common in both the spleen and brain tissues. These cells had much higher levels of exhaustion markers like Lag3 and CTLA-4. This was accompanied by an increase in the transcription factor EOMES, which is a key regulator of exhausted T cells and is very important for changing how T cells develop (7, 38). Slamf 7 is very present in the terminal branch of DNT, which makes it a good marker for terminal exhaustion. Slamf 7 has been shown to control T cell metabolic reprogramming. When it is overexpressed, it stops AMPKα phosphorylation, which stops fatty acid oxidation and speeds up glycolysis. Abnormal glycolysis upregulates the IL-17 transcription factor RORγt, leading to increased IL-17 secretion and worsening central inflammation in autoimmune diseases (39, 40). DNT in NPSLE have special functions that are different from Tex cells, which are completely inactive in tumours. They keep or even boost the release of proinflammatory substances like IL-17 and IL-4. The chemokine receptor Gpr183 and the adhesion molecule SELL are highly expressed, which lets them get into the central nervous system through the “splenic differentiation → haematogenous migration → blood-brain barrier crossing” pathway (39).

## 4 Tex-related biomarkers in SLE: bridging phenotype, disease activity, and prognosis

Biomarkers associated with T cell exhaustion in SLE are essential for connecting Tex phenotypes, disease activity and clinical prognosis. They allow for exact measurements of the characteristics and functional states of Tex subpopulations, while also showing how the disease is progressing, how severe the immune dysregulation is and how well the treatment is working. These biomarkers offer objective quantitative instruments for stratified management, therapeutic efficacy evaluation and prognosis determination in SLE patients, whilst also facilitating the elucidation of the molecular mechanisms governing Tex.

### 4.1 Causal association between blood-detectable biomarkers and disease state

A recent study analysed peripheral blood samples from SLE patients in the Gene Expression Omnibus (GEO) and found that four type I interferon-stimulated genes (ISGs)—MX1, LY6E, IFI44, and OASL—show different expression levels in the blood of people with SLE. This is closely linked to the infiltration of resting memory CD4^+^T cells. The depletion of resting memory CD4^+^T cells, which are important for keeping immune memory, may cause problems with the immune system. This implies that the biomarkers may be involved in the depletion process by modulating this cell population. Biomarker expression exhibited substantial positive correlations with three fundamental immune pathways—namely, the NOD-like, Toll-like, and RIG-I-like receptor signalling pathways—all of which are essential in the pathogenesis of systemic lupus erythematosus. The expression levels of these four genes are closely linked to the activity of SLE, such as nephritic lupus activity and proteinuria (**Table 1**). The researchers also checked peripheral blood from 6 SLE patients and 6 healthy controls using RT-qPCR, and their analysis confirmed that these biomarkers have clear diagnostic value for SLE (11, 41–43).

**Table 1:** Tex-related SLE biomarkers (11).

Marker name	Relationship with SLE
MX1	Highly expressed in peripheral blood of lupus nephritis (LN) patients, serving as a sensitive indicator of treatment response in LN.
LY6E	Its expression in splenic lymphocytes of lupus mice correlates positively with disease severity and is upregulated in proteinuria-induced renal proximal tubules, contributing to renal injury.
IFI44	A specific marker that distinguishes active from inactive LN.
OASL	Very high levels in peripheral blood mononuclear cells (PBMCs) and CD19^+^B cells from people with active LN.

### 4.2 Progressive association of TUTase with patient subtypes

A recent study identified four core terminal uridylyltransferases (ZCCHC11, PAPD5, ZCCHC6, MTPAP) from GEO, enzymes primarily catalysing 3’-RNA uridylation during transcription, and confirmed their association with T cell exhaustion. Based on these four core terminal uridylyltransferases, SLE patients were classified into three subtypes (Clusters A-C). Subtype characteristics showed progressive relationships with Tex expression, immune infiltration, disease activity, and prognosis. This enables personalised assessment of Tex-associated features and disease activity, facilitating more precise patient stratification (**Table 2**) to predict disease activity and guide immunotherapy (12, 44).

**Table 2:** Three subtypes of SLE patients (12).

Subtype	Core features (immune infiltration + Tex expression)	SLEDA (disease activity)	Prognosis
Cluster A	1. Immune infiltration is intermediate between B and C (no clear innate/adaptive immune advantage); 2. Moderate expression of Tex markers.	Moderate (between B and C)	Moderate prognosis: Immunologically balanced state with relatively stable disease activity.
Cluster B	1. Enriches innate immune cells (activated dendritic cells, NK cells, macrophages, plasma cell-like dendritic cells); 2.Shows very little expression of Tex markers (significantly lower transcriptional levels of inhibitory receptors like PD-1, LAG-3, TIGIT and CTLA-4).	Highest (median significantly higher than A and C, P<0.001)	Worst prognosis: Innate immune overactivation + Tex deficiency, leading to worsened immune damage
Cluster C	1. Enrichment of adaptive immune cells (activated CD4^+^ T cells, CD8^+^ T cells, B cells); 2. Highest expression of Tex markers (significantly elevated transcriptional levels of inhibitory receptors)	Lowest (median below Group A, though no statistically significant difference, but with more stable clinical activity)	Best prognosis: Adaptive immune cells exhibit significant Tex activity, capable of suppressing excessive autoimmune responses.

PDCD1 (PD-1), HAVCR2 (TIM-3), LAG3, TIGIT, BTLA, CTLA4 and other Tex markers are all well-known molecular markers of Tex.

## 5 Therapeutic strategies addressing T cell exhaustion: accurate regulation grounded in bidirectional effects

**Figure 2:**
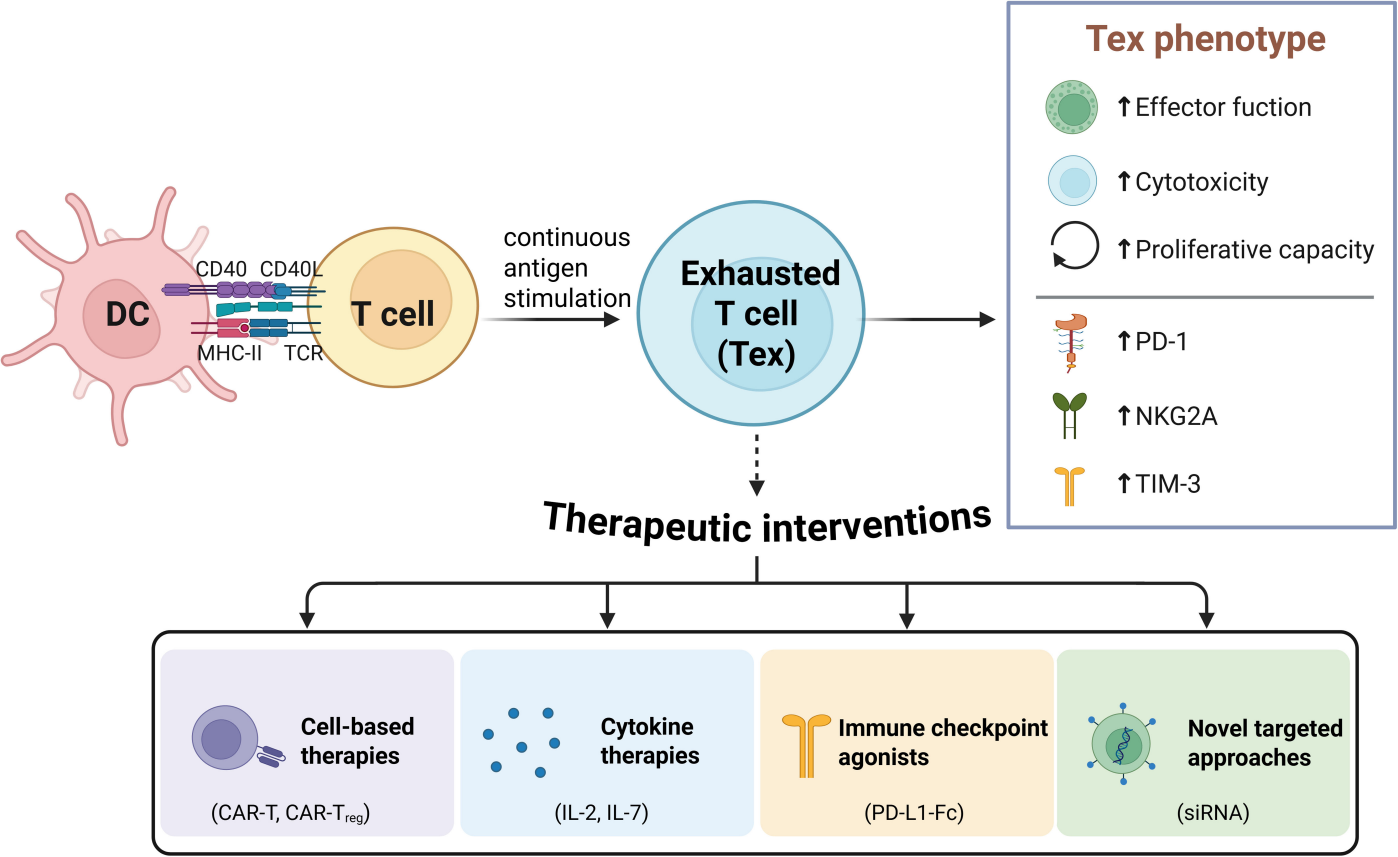
Regulatory Mechanisms and Therapeutic Strategies for Tex in SLE.

Autoimmune diseases, including SLE, are caused by abnormal responses of the adaptive immune system. Current therapies such as biologic bDMARDs encounter obstacles such as inadequate response rates, challenges in sustaining remission, and considerable adverse effects. Some patients exhibit no response to conventional immunosuppressants and are unable to attain drug-free remission, necessitating the urgent development of innovative therapeutic strategies (2, 3). To deal with the dual role of Tex in SLE—both protective and pathogenic—therapeutic strategies should focus on boosting protective exhaustion whilst lowering pathogenic exhaustion. This entails the precise regulation of Tex subset functionality, the localised immune microenvironment, and critical molecular signalling pathways. These methods try to restore immune balance without causing immune dysregulation (**Figure 2**).

### 5.1 Clinically established or emerging strategies to enhance protective exhaustion

#### 5.1.1 Optimising IL-2 strategies

Patients with SLE show fewer or less functional regulatory T cells, and IL-2 is an important cytokine for Treg differentiation and survival. Low-dose IL-2 (IL-2LD) therapy has been increasingly utilised to activate Tregs in autoimmune conditions (45, 46). To tackle problems like IL-2’s short natural half-life, new formulations are being created. These include IL-2 variants, IL-2-anti-IL-2 complexes, and immunomodulatory cytokines. Rezpegaldesleukin (NKTR-358) is one example. It is meant to lower off-target activation and stop triggers of exhaustion (47–51). Among the currently available approaches, low-dose IL-2 therapy represents one of the most clinically advanced strategies and has already been evaluated in several early-phase clinical studies in patients with SLE (52).

#### 5.1.2 Immune checkpoint agonists

Immune checkpoint agonism remains largely at the preclinical stage in SLE. Evidence supporting this strategy is primarily derived from lupus-prone mouse models, where enhancement of PD-1 signalling through PD-L1-Fc fusion proteins has shown renoprotective effects. This regulated the balance of the immune microenvironment, significantly reducing proteinuria and renal injury. Traditional LN treatments primarily rely on glucocorticoids and immunosuppressants such as cyclophosphamide and mycophenolate mofetil, but these approaches carry significant side effects, high recurrence rates, and inadequate renal protection. This strategy demonstrates distinct advantages in both mechanism and efficacy by enhancing protective exhaustion and improving the pathological state of LN (36, 53).

#### 5.1.3 CAR-Treg therapy

CAR-Treg therapy is currently considered an exploratory cellular immunotherapy. Although encouraging results have been obtained in preclinical lupus models, clinical evidence in SLE remains limited. In CAR-Treg, Tregs are genetically modified to express CARs that target autoantigens like CD19 or Smith antigen. This method brings the body back to its normal state by stopping effector immune cells instead of getting rid of them. For example, Tregs that express CD19 can target B cells by releasing IL-10 and TGF-β to stop B-cell activation and cause effector T-cell apoptosis through Fas/FasL. In humanised SLE mouse models, CD19 CAR-Tregs diminished autoantibody levels and ameliorated renal injury. This approach is currently in preclinical stages and could be a way to precisely restore tolerance (54–56). Preclinical studies show that CAR-Tregs can keep working in autoimmune diseases like type 1 diabetes and multiple sclerosis, which indirectly shows that they can help prevent exhaustion and give new ideas for treating SLE (57).

### 5.2 Suppressing pathogenic exhaustion—reverse regulation

#### 5.2.1 Multi-antigen targeted CAR-T

In SLE, pathogenic B cells and autoreactive T cells keep the immune system activated and cause tissue damage. Traditional CAR-T strategies usually target a single B-cell antigen (like CD19), which can be limited because they might not completely eliminate the pathogenic cells and the ongoing presence of autoreactive clones could continue to drive immune imbalance. To tackle this, researchers are exploring multi-antigen-targeted CAR-T designs in mouse models of autoimmune disease. For example, dual-target or tandem CAR-T constructs that aim at both CD19 and CD20 on autoreactive B cells can boost the clearance of pathogenic cell populations, cut down on autoantibody production, and ease T cell overactivation, potentially preventing further exhaustion of CD8^+^ T cells. Although evidence in SLE mainly comes from preclinical studies so far, these strategies show a conceptual framework for controlling multiple types of pathogenic immune cells whilst keeping immune balance (54, 58–60).

#### 5.2.2 Novel targeted therapies

5-Methylcytosine modification (m5C) is an important way to control gene expression after transcription. A comprehensive analysis of single-cell transcriptomics and m5C epigenomic sequencing (m5C-seq) has identified NOP2/Sun RNA methyltransferase 4 (NSUN4) as a pivotal regulator in the pathogenesis of SLE. Knocking out NSUN4 lowers the level of CD74 by lowering m5C levels and stops CD8^+^T cells from getting tired by stopping mitosis through the CD44/mTOR pathway (mechanistic target of rapamycin). Research showed that siRNA delivered by nanoparticles that targets NSUN4 reduces CD8^+^Tcell exhaustion by controlling m5C modification and mitochondrial autophagy. This helps prevent autoimmune kidney damage in a mouse model of SLE (61). Transient anti-TCRβ monoclonal antibody treatment also raises the levels of PD-1 and TIM-3 in CD4T^+^cells whilst lowering the levels of IFN-γ. This shows that the cells are exhausted, which helps SLE mice live longer (57).

#### 5.2.3 Cytokine therapy

Cytokine-based therapies offer a promising approach to regulating T cell exhaustion in patients with SLE, working by restoring immune balance rather than causing broad immunosuppression. Cytokines like IL-2, IL-7, IL-15, and IL-21 are core components of this therapy (62–66). Moreover, IL-10 and IL-12 have shown potential in preclinical studies to enhance the effectiveness of adoptive T cell therapy and immune checkpoint inhibition (67, 68). Although direct evidence in SLE is still limited, these cytokines may influence the differentiation and maintenance of exhausted T cell subsets, making them potential therapeutic targets for future research. The latest advances in cytokine engineering further expand treatment possibilities. Genetically modified IL-2 variants and orthogonal IL-2/IL-2 receptor systems can selectively stimulate target T cell populations (69, 70). Compared to traditional cytokine administration methods, these approaches allow for more precise immune modulation.

### 5.3 Contradictions and balancing principles in treatment strategies

Studies show that the PD-1/PD-L1 pathway is the main signalling pathway that connects tired CD8^+^T cells to activated memory B cells. Its dysfunction substantially exacerbates the immune dysregulation in SLE. In people with SLE, tired PD-1-high CD8^+^T cells can’t stop activated memory B cells from growing, and B-cell surface PD-L1 expression drops, which starts a cycle of “T-cell exhaustion-B-cell overactivation.” PD-1 agonists only bring back the function of tired CD8^+^T cells. Genetic transfection (for example, lentivirus-mediated PD-L1 overexpression) or drug interventions (for example, finding small molecules that cause PD-L1 expression) can raise the levels of PD-L1 in B cells. Or using anti-CD20 monoclonal antibodies such as rituximab to get rid of PD-L1-low B cells that are active, which stops them from making CD8^+^ T cells work. This indirectly brings exhausted CD8^+^ T cells back to life and makes them able to healthy subjects IFN-γ again (71).

Immune checkpoint blockade (ICB) therapy necessitates prudence in autoimmune diseases; although PD-1/PD-L1 blockade in cancer can mitigate T-cell exhaustion and augment antitumour immunity, excessive reversal of exhaustion in SLE may intensify immune dysregulation and exacerbate autoimmune damage. This could be because T cell exhaustion in cancer mostly affects T cells that are specific to tumour antigens, whilst in autoimmune diseases, exhausted T cells include both auto-reactive and protective T cells. Non-specific blockade activates both types of cells at the same time, throwing off the balance of the immune system.

## 6 Conclusions

Tex plays a dual role in SLE, limiting autoreactive T cell activity whilst potentially contributing to immune dysfunction. Recognition of Tex in autoimmune disease has expanded insights beyond infection and cancer, revealing novel pathogenic mechanisms and therapeutic opportunities.

However, findings in SLE are sometimes conflicting: some studies suggest protective roles for Tex, whilst others indicate pathogenic effects, reflecting differences in patient populations, disease stages, tissue compartments and Tex definitions. Most evidence derives from transcriptomic analyses, animal models or small observational cohorts, with few mechanistic or prospective clinical studies. The dynamic evolution of Tex subsets during disease flares, remission and treatment remains poorly understood.

Translating Tex-targeted therapies to SLE presents challenges. Reversing exhaustion may restore protective function but risk reactivating autoreactive T cells, whereas enhancing exhaustion could compromise host defence. Future research should focus on longitudinal human studies, standardised Tex subset definitions, biomarker-guided patient stratification and selective modulation of exhausted.
